# Prostaglandin E_**2**_ Does Not Modulate CCR7 Expression and Functionality after Differentiation of Blood Monocytes into Macrophages

**DOI:** 10.1155/2013/918016

**Published:** 2013-11-05

**Authors:** Marc-André Allaire, Bérengère Tanné, Sandra C. Côté, Nancy Dumais

**Affiliations:** Département de Biologie, Faculté des Sciences, Université de Sherbrooke, Sherbrooke, QC, Canada J1K 2R1

## Abstract

Previously, we demonstrated that prostaglandin E_2_ (PGE_2_) induces C-C chemokine receptor type 7 (CCR7) expression on human monocytes, which stimulates their subsequent migration in response to the CCR7 natural ligands CCL19 and CCL21. In this study, we determined whether PGE_2_ affects CCR7 expression on macrophages. Flow cytometric analysis and chemotaxis assays were performed on Mono Mac-1-derived macrophage (MDMM-1) as well as unpolarized monocyte-derived macrophages (MDMs) to determine the CCR7 expression and functionality in the presence of PGE_2_. Data revealed that a MDMM-1 exhibited markedly downregulated CCR7 expression and functionality that were partially restored by treatment with PGE_2_. In MDMs, we observed a drastic downregulation of CCR7 expression and functionality that were unaffected following PGE_2_ treatment. Our data indicate that monocyte differentiation induces the loss of CCR7 expression and that PGE_2_ is unable to modulate CCR7 expression and functionality as shown previously in monocytes.

## 1. Introduction

Monocytes and macrophages orchestrate proper immune responses to pathogens. Monocytes have been demonstrated to be precursors of professional antigen-presenting cells, such as macrophages and dendritic cells (DCs) [1–3]. In response to danger stimuli, circulating blood monocytes migrate into damaged or infected tissues and differentiate into mature macrophages or DCs. After taking up antigens, the activated macrophages and DCs migrate to the draining lymph nodes to present the antigens to T and B cells. 

Chemokine receptors confer upon cells the ability to detect and move directionally toward a chemotactic stimulus. C-C chemokine receptor type 7 (CCR7) plays a leading role in the mechanism controlling the entry of lymphocytes and mature DCs into lymph nodes. Within lymph nodes, these cells encounter other immune cells for activation, determining the success of cellular immunity after infection [4]. For immature DCs, the maturation process is initiated upon sensing “danger signals” (tissue damage, inflammatory cytokines, or pathogens) [5]; this process occurs concomitantly with their migration from peripheral tissues to the draining lymph nodes. During maturation, CCR7 expression is upregulated, which guides the migratory DCs to the lymph nodes [6–8]. The chemokines CCL19 and CCL21 are the natural ligands of CCR7, and they are expressed by lymphatic endothelium and/or within lymph nodes by stromal cells, endothelial cells, and DCs [9–13]. Mice deficient in CCL19, CCL21, or CCR7 demonstrate defective DC trafficking and altered immune responses [6, 14, 15].

Recently, CCR7 expression was discovered on human monocytes [16]. As observed with DC migration [17], prostaglandin E_2_ (PGE_2_), a pleiotropic immunomodulatory molecule, appears to have a dual role in monocyte migration by regulating the expression and activity of CCR7 through the involvement of EP_2_ and EP_4_ receptors [16]. Moreover, in DCs, PGE_2_ has been reported to synergize with tumor necrosis factor-alpha (TNF-*α*) to promote CCR7 expression and the chemotactic responsiveness of DCs to CCL19 and CCL21 [17–19]. Recent results indicate that CCL19 binding to CCR7 promotes the activation of p38, extracellular signal-regulated kinase 1/2, and c-Jun N-terminal kinase and leads to monocyte migration. Moreover, the RhoA/ROCK pathway is essential for PGE_2_-mediated CCR7-dependent monocyte migration [20]. The physiological importance of CCR7 for the immune response has been demonstrated by several studies, and although CCR7 expression and functionality have been extensively characterized in DCs, much less is known about the fate of its expression in macrophages.

As it has been demonstrated that monocytes express the chemokine receptor CCR7 and differentiate upon immune activation, we asked whether monocyte maturation in the presence of PGE_2_ has a direct effect on CCR7 expression and functionality. In a unpolarized environment, our results demonstrate that monocyte maturation downregulates CCR7 expression in macrophages derived from Mono Mac-1 cells (MDMM-1 cells) and monocyte-derived macrophages (MDMs) from healthy donors. In parallel, chemotaxis assays were performed to establish the influence of differentiation on CCR7 signaling function. Our results clearly indicate that MDMM-1 cells and MDMs lose functional CCR7 expression, which inhibits their migration toward the CCR7 ligands CCL19 and CCL21. Thus, these data reveal that unlike DCs, macrophages lose CCR7 expression and functional activity upon differentiation, even in the presence of PGE_2_.

## 2. Materials and Methods

### 2.1. Reagents

PGE_2_ was purchased from Sigma Aldrich (Oakville, ON, Canada). The chemokines CCL19 and CCL21 and antibodies anti-CCR7-APC and anti-IgG2a-APC were purchased from R&D Systems (Minneapolis, MN, USA). Anti-CD14-FITC, anti-CD64-FITC (BD Biosciences, Mississauga, ON, Canada), and anti-IgG2a-FITC were purchased from Santa Cruz Biotechnology (Santa Cruz, CA, USA).

### 2.2. Cell Culture

The monocytoid cell line Mono Mac-1 was purchased from the German Collection of Microorganisms and Cell Culture (Braunschweig, Germany). Cells were maintained in RPMI 1640 medium (Wisent, Saint-Bruno, QC, Canada) supplemented with 10% heat-inactivated fetal bovine serum (FBS; Wisent), 1 mM sodium pyruvate (Wisent), 0.1 mM nonessential amino acids, 100 U/mL penicillin G, and 100 *μ*g/mL streptomycin (Wisent). Mono Mac-1 cells were induced to differentiate into macrophage-like (MDMM-1) cells with PMA. Briefly, cells were plated in 100 mm dishes (7 × 10^6^ cells) in fresh medium and incubated with 20 ng/mL PMA for 72 h [21]. Mono Mac-1 and MDMM-1 cells were stimulated for the indicated times using 1 *μ*M PGE_2_.

### 2.3. Blood Monocyte Isolation and MDM Differentiation

Total blood mononuclear cells were isolated from the blood of healthy donors using lymphocyte separation Medium 1077 (Sigma) and washed twice in Hank's balanced salt solution (Wisent). Cells were cultured in RPMI 1640 medium, 20% heat-inactivated FBS, and 10% heat-inactivated human serum for 2 h before use. Monocytes were washed from nonadherent cells with phosphate-buffered saline (PBS). Monocytes were then enriched from peripheral blood mononuclear cells using the MACS Monocyte Isolation Kit II and MACS LS Columns (Miltenyi Biotec, Auburn, CA, USA), yielding an average purity of 98%. The purity was assessed by flow cytometric analyses as recommend by the manufacturer, and isolated monocytes were fluorescently stained with CD14-FITC and anti-Biotin-PE that labeled nonmonocytes. To differentiate monocytes into MDMs, cells (2 × 10^6^/mL) were plated in RPMI 1640 medium with 2 mmol/L l-glutamine (Wisent) containing 20% human AB serum (Wisent) in 24-well plates (Millipore, Nepean, ON, Canada). After 24 h, nonadherent cells were removed, and adherent cells were cultured in RPMI with 20% FBS (Wisent) in 5% CO_2_ at 37°C for 12 days. Medium was refreshed every 4 days. Macrophages were detached using Cell Dissociation Buffer (Wisent), washed with 1× PBS, and used in experiments. Blood monocytes and MDMs were stimulated for the indicated times using 1 *μ*M PGE_2_.

### 2.4. Microscopy

Images of whole-cell morphology were acquired by interferential contrast using a Olympus IX70 microscope (Olympus Canada Inc., Richmond Hill, ON, Canada) and processed by Image Pro Plus software (Media Cybernetics Inc., Bethesda, MD, USA).

### 2.5. Flow Cytometry

Cells were collected and washed in 1× PBS. Fc receptors were blocked using 1 *μ*g of purified IgG (Santa Cruz Biotechnology) for 15 min at room temperature and washed in PBS containing 3% bovine serum albumin (BSA). Cells were labeled with an anti-CCR7 antibody conjugated with allophycocyanin or the corresponding antibody isotype for 45 min on ice and protected from light. Cells were then washed three times with PBS containing 3% BSA. Samples were analyzed in a BD FACSCalibur flow cytometer (BD Biosciences) using CellQuest software (BD Biosciences).

### 2.6. Chemotaxis Assay

Chemotaxis was measured by migration through a polycarbonate filter with 5 and 8 *μ*m pores for blood cells and Mono Mac-1 cells, respectively, in 96-well Transwell chambers (Millipore). RPMI with 0.25% BSA (100 *μ*L) containing the indicated concentrations of CCL19 or CCL21 or medium alone as a control for spontaneous migration was added to the lower chamber. To the upper chamber, 1 × 10^5^ cells (150 *μ*L) were added. Monocytes and macrophages were then incubated for 4 h at 37°C. A 150 *μ*L aliquot of the cells that migrated to the bottom chamber was counted by flow cytometry in a FACScan that acquired events for a fixed period of 60 s using CellQuest software. Spontaneous migration was subtracted to calculate the specific migration. The percentage of migrated cells was calculated as follows: the number of migrated cells in response to medium only was subtracted from the number of migrated cells in medium supplemented with CCL19 or CCL21, and this number was reported according to the total input of cells. Each experiment was performed in triplicate, and migration assays were repeated at least three times. 

### 2.7. Reverse Transcription (RT) and Real-Time Polymerase Chain Reaction (PCR)

MDMM-1 cells were incubated with or without 1 *μ*M PGE_2_ for the indicated times, and total RNA was extracted with the Total RNA Kit E.Z.N.A. (Omega Bio-Tek, Mississauga, ON, Canada) according to the manufacturer's instructions. RNA was reverse-transcribed into cDNA in the presence of 200 U of M-MLV RT (Promega, Madison, WI, USA), 0.5 *μ*g of oligonucleotide d(T)_15_, and 500 *μ*M deoxyribonucleotide triphosphates at 42°C for 1 h. One microliter of cDNA was used and analyzed using the Platinum SYBR Green qPCR SuperMix System from Invitrogen (Burlington, ON, Canada). The PCR mixture consisted of 0.25 *μ*M forward and reverse primers for CCR7 as described previously [16], 0.4 mM deoxyribonucleotide triphosphate, 2 mM MgCl_2_, and 1.25 U of Taq DNA polymerase (Roche, Indianapolis, IN, USA). The PCR cycling conditions consisted of initial denaturation at 95°C for 10 min and 40 cycles of 95°C for 30 s, 55°C for 45 s, and 72°C for 45 s. Samples were run in an Applied Biosystems 7500 gel electrophoresis apparatus (Applied Biosystems, Foster City, CA, USA), and the results were analyzed using v1.4 software. 

### 2.8. Statistical Analysis

Each experiment was performed at least three times. Statistically significant differences between experimental groups were evaluated using a paired *t*-test. Computations were performed using GraphPad PRISM version 5.0b statistical software.

## 3. Results and Discussion

### 3.1. Surface Expression of CCR7 on Human Monocytes Is Modulated during Differentiation

**Figure 1:**
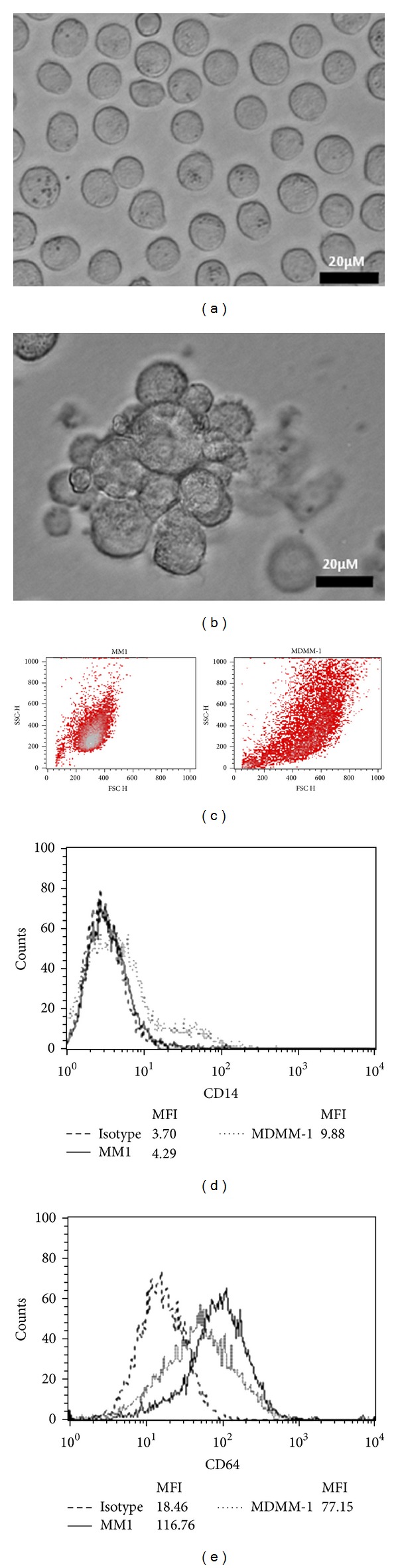
Differentiation of Mono Mac-1 cells into MDMM-1 cells. Representative interferential contrast images of Mono Mac-1 (a) and MDMM-1 (b) cells after 72 h of differentiation with 20 ng/mL PMA, as well as forward light scatter and side light scatter plots (c). CD14 (d) and CD64 (e) expression was analyzed by flow cytometry before and after differentiation. Data shown are representative of three experiments.

Mono Mac-1 is a cell line assigned to the monocytic lineage according to morphological, cytochemical, and immunological criteria [22]. Recently, Mono Mac-1 cells and blood-isolated monocytes were shown to express functional CCR7 chemokine receptors that are upregulated by treatment with PGE_2_ [16]. In this study, we investigated the impact of the maturation of monocytes into macrophages on CCR7 expression and functionality. First, the differentiation of Mono Mac-1 cells into a more mature, macrophage-like phenotype was induced by treatment with 20 ng/mL PMA for 72 h as previously described [21]. Differentiation was assessed by morphological changes such as cell clustering, cellular adhesion to the bottom surface of the culture dish, and a reduction in the nucleocytoplasmic ratio due to an increase in cytoplasmic volume [23, 24]. As anticipated and shown in Figures 1(a) and 1(b), MDMM-1 cells exhibited increased cytoplasmic volume compared to that in Mono Mac-1 cells. Moreover, we observed that PMA treatment enhanced the clustering of MDMM-1 cells. Another feature of macrophage differentiation is enhanced granularity, as demonstrated by increased side scatter (SSC) on flow cytometry [25]. Compared to Mono Mac-1 cells, MDMM-1 cells have increased SSC as shown in Figure 1(c). As CD14 expression is a phenotypic marker for monocyte differentiation, CD14 expression on Mono Mac-1 cells was determined after differentiation using PMA. The percentage of CD14^+^ cells increased from 2.1% to 12.6% during maturation, whereas the mean fluorescence intensity (MFI) was 4.3 and 9.9 in Mono Mac-1 and MDMM-1 cells, respectively (Figure 1(d)). In addition, it has been revealed that the expression of the cell surface antigen CD64 is downregulated in macrophages [26]. Thus, we next investigated whether macrophages exhibit a significant decrease in CD64 expression, and the results indicated that CD64 expression was significantly decreased in MDMM-1 cells (34.6% compared to 68.9% in Mono Mac-1) (Figure 1(d)). 

**Figure 2:**
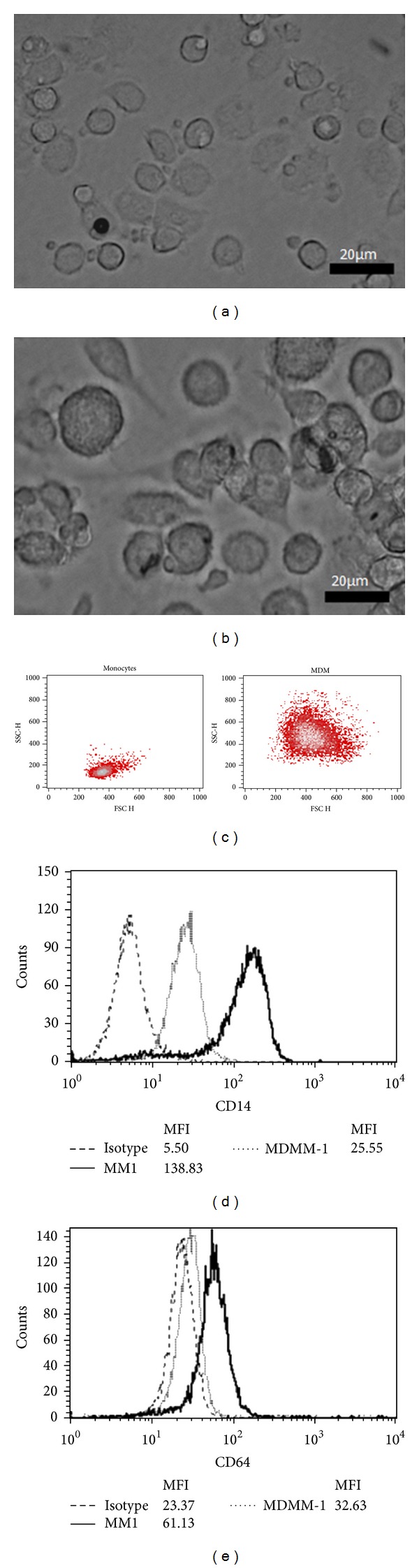
Differentiation of freshly isolated blood monocytes into MDMs. Representative interferential contrast images of blood monocytes (a) and MDMs (b) after 8 days of differentiation in culture, as well as forward light scatter and side light scatter plots (c). CD14 (d) and CD64 (e) expression was analyzed by flow cytometry before and after differentiation. Data shown are representative of three experiments.

We also investigated whether monocytes from the blood of healthy donors that were differentiated into MDMs exhibited the morphological and immunological characteristics of macrophages. As observed with Mono Mac-1 and MDMM-1 cells, morphological differences were noted between monocytes and MDMs (Figures 2(a) and 2(b)), and SSC also increased in macrophages (Figure 2(c)). In human blood cells, monocyte differentiation into macrophages resulted in significantly decreased CD14 expression [27]. We found that macrophage differentiation decreased CD14^+^ expression (MFI of 25.6 in MDMs; MFI of 138.9 in blood monocytes). Conversely, MDMs exhibited a significant diminution in CD64 expression as expected (MDMs: 4.9% with a MFI of 32.7; blood monocytes: 31.0% with a MFI of 61.1) (Figure 2(d)). Altogether, our results demonstrate that MDMM-1 cells and MDMs exhibit characteristics attributed to macrophages in the literature.

**Figure 3:**
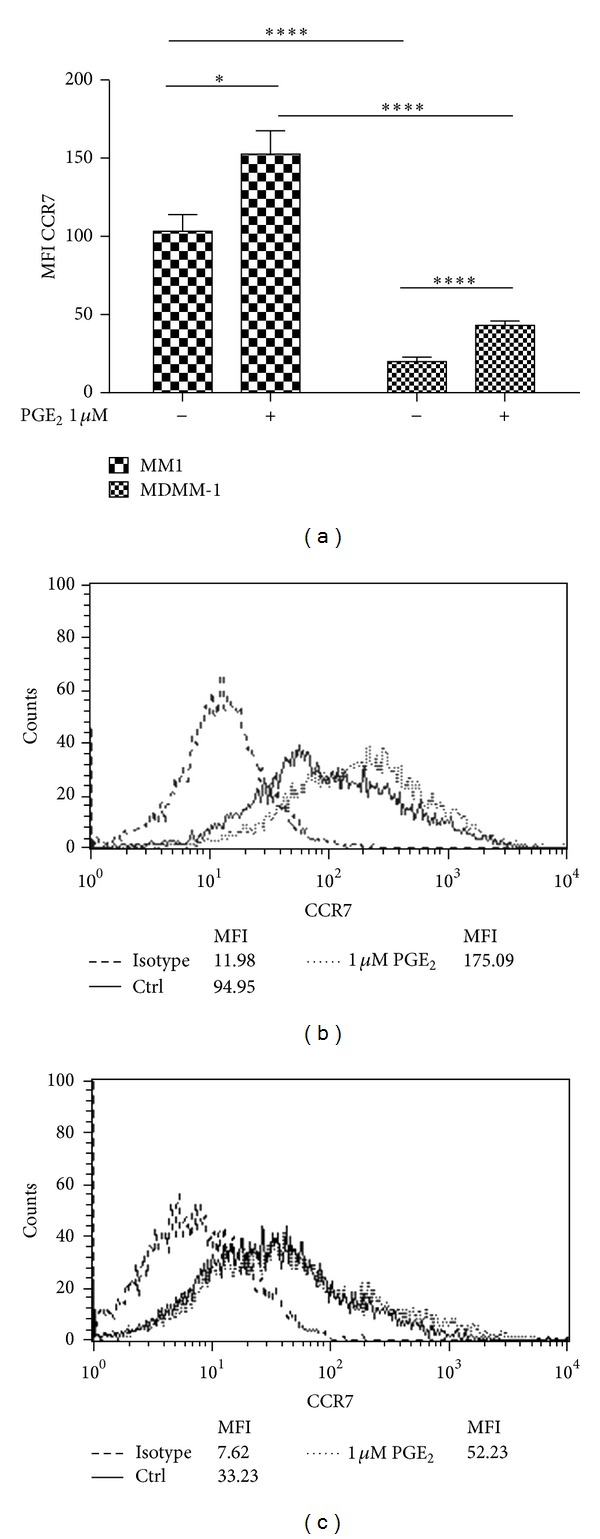
Differentiation of Mono Mac-1 cells into MDMM-1 cells induces the loss of CCR7 expression. Mono Mac-1 cells were differentiated in MDMM-1 cells as described. (a) CCR7 surface expression was evaluated by flow cytometry on Mono Mac-1 and MDMM-1 cells following treatment with 1 *μ*M PGE_2_ for 24 h. Data are the mean of four representative experiments, and representative histograms obtained for Mono Mac-1 (b) and (c) MDMM-1 cells are presented. *P < 0.5, **P < 0.005, ***P < 0.001, and ****P < 0.0001.

**Figure 4:**
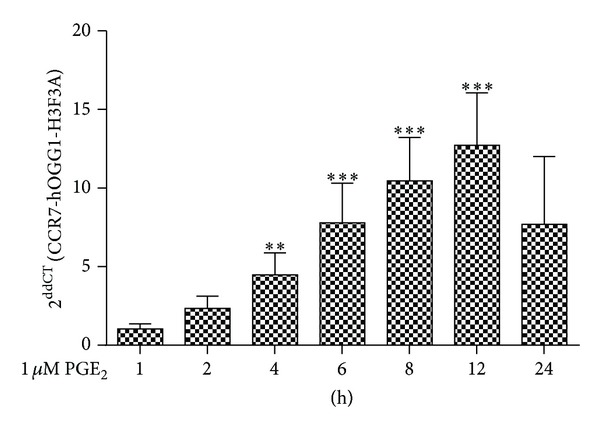
Stimulation with PGE_2_ induces the upregulation of CCR7 mRNA on MDMM-1 cells. Cells were stimulated with 1 *μ*M PGE_2_ for the indicated times. Total RNA was extracted and submitted to real-time PCR to detect the presence of CCR7 mRNA. Data represent the means of four different experiments. **P < 0.005, ***P < 0.001.

To evaluate whether monocyte maturation in the absence of M1/M2 polarizing conditions affects the cell surface expression of CCR7 on Mono Mac-1 and MDMM-1 cells, the cells were incubated in the presence or absence of PGE_2_ for 24 h, and the expression of CCR7 was analyzed by flow cytometry (Figure 3). Increased cell surface expression of CCR7 has been previously demonstrated on Mono Mac-1 cells by Côté et al [16]. However, an important diminution of CCR7 expression is detected on cell surface of MDMM-1 cells (Mono Mac-1: a MFI of 94.95; MDMM-1: a MFI of 52.23). As it has been demonstrated that PGE_2_ increases CCR7 expression on Mono Mac-1 cells, we next determined whether PGE_2_ stimulation also affected CCR7 expression on MDMM-1 cells. After 24 h of treatment, CCR7 expression was significantly increased on MDMM-1 cells (MFI of 52.23). We asked whether CCR7 upregulation was also observed at the mRNA level by using real-time RT-PCR to verify the presence of CCR7 mRNA after Mono Mac-1 differentiation and treatment with PGE_2_ over a 24 h time course (Figure 4). Our results demonstrated that CCR7 mRNA expression is modulated by PGE_2_ in MDMM-1 cells. As observed in undifferentiated Mono Mac-1 cells, CCR7 mRNA levels were highest when MDMM-1 cells were stimulated with 1 *μ*M PGE_2_ for 12 h. Altogether, our results indicate that PMA-induced Mono Mac-1 differentiation decreased the cell surface expression of CCR7, but treatment with the immunomodulatory molecule PGE_2_ slightly increased the transcription and expression of CCR7 in MDMM-1 cells.

**Figure 5:**
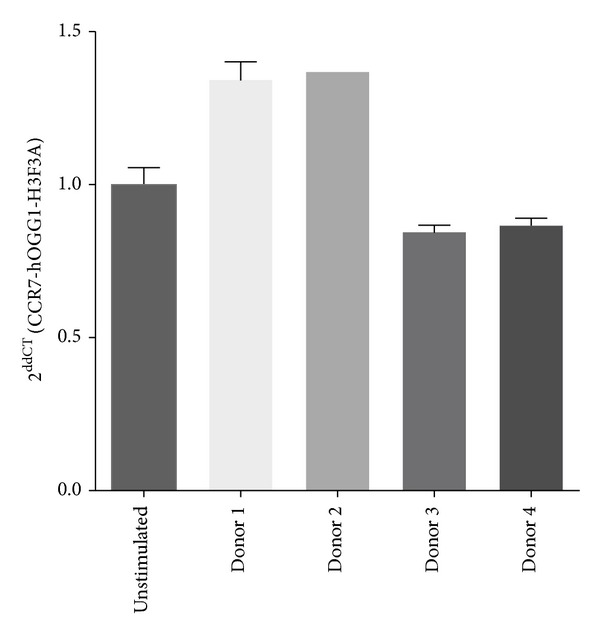
PGE_2_ does not modulate CCR7 mRNA transcription in MDMs. MDMs were obtained from monocytes from four different healthy donors and were stimulated with 1 *μ*M PGE_2_ for 6 h. Total RNA was extracted and submitted to real-time PCR to detect the presence of CCR7 mRNA. Results shown are the means (±SD) of four determinations.

**Figure 6:**
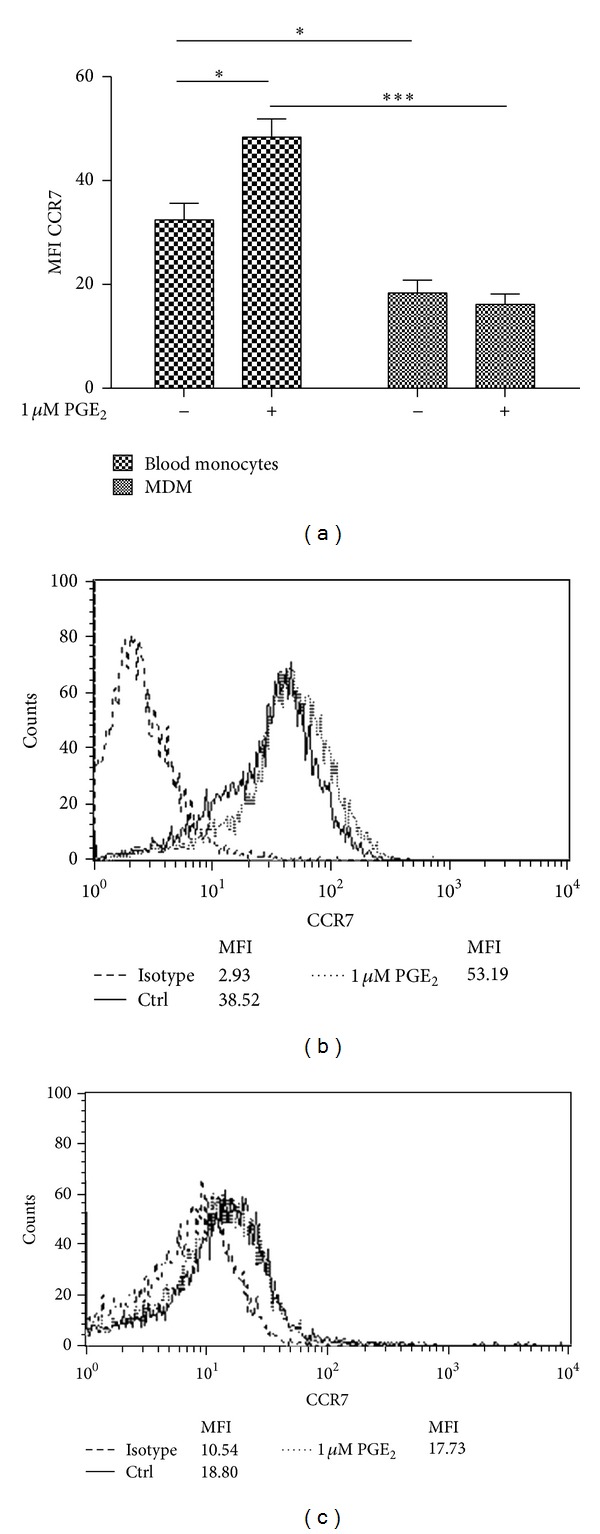
PGE_2_ has no effect on MDMs CCR7 expression. (a) CCR7 surface expression on blood monocytes and MDMs was evaluated by flow cytometry following treatment with 1 *μ*M PGE_2_ for 24 h. Data represent the means of five different healthy donors. Histograms from one of five experiments for (b) blood monocytes and (c) MDMs are presented. *P < 0.5, ***P < 0.001.

To further investigate the effects of monocyte maturation on CCR7 expression, we repeated this series of experiments with freshly isolated human blood monocytes. Monocytes were cultured up to 8 days to induce differentiation, and CCR7 mRNA level (Figure 5) as well as CCR7 cell surface expression was analyzed by flow cytometry (Figure 6). As observed in MDMM-1 cells, CCR7 expression was higher on human blood monocytes, and it was downregulated upon the maturation of MDMs (human blood monocytes: 14.0% with a MFI of 31.0; MDMs: 4.9% with a MFI of 16.7). Interestingly, and unlike the effect observed in the differentiated MDMM-1 cell line, no significant change in CCR7 mRNA level and CCR7 expression was detected after PGE_2_ treatment, although a trend toward lower CCR7 expression was observed. Thus, overall the differentiation of cultured monocytes or freshly isolated monocytes from human blood resulted in a decrease in CCR7 surface expression, and PGE_2_ slightly restored CCR7 expression in MDMM-1 cells but not MDMs.

### 3.2. MDMs Do Not Migrate in Response to CCR7 Ligands

**Figure 7:**
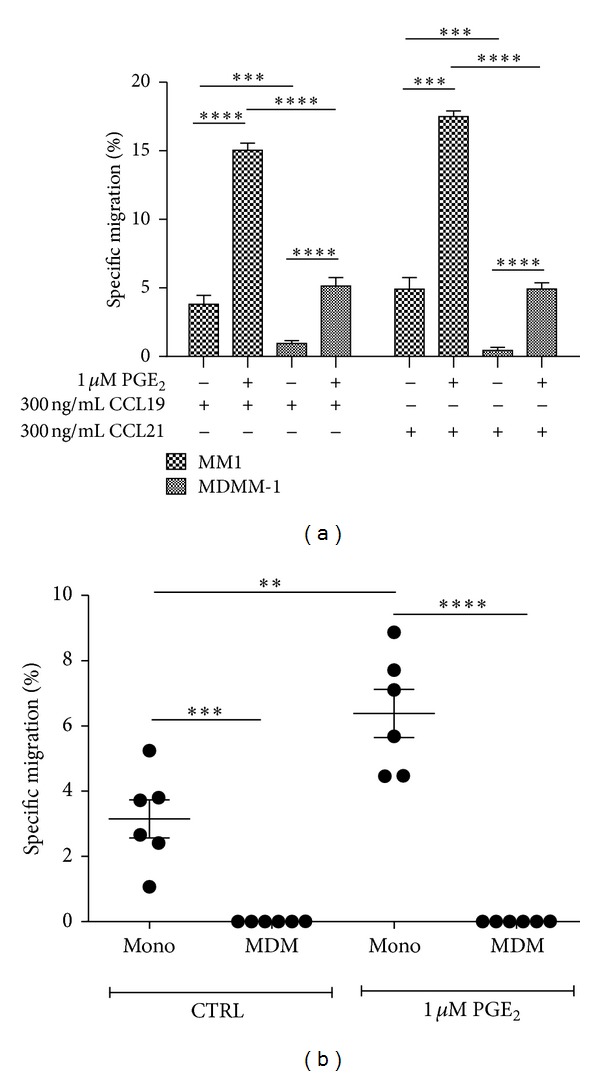
MDMM-1 cells and MDMs do not migrate toward the CCR7-specific ligands CCL19 and CCL21. (a) Chemotaxis assays using 300 ng/mL CCL19 and CCL21 were performed using MM1 and MDMM-1 cells incubated in the presence or absence of 1 *μ*M PGE_2_ for 24 h. Data represent the means of three different experiments. (b) Blood monocytes from five different healthy donors were differentiated into MDMs and used for chemotaxis assays as described. The mean number of spontaneously migrated cells was subtracted from the number of cells that migrated in response to chemokines. **P < 0.01,  ***P < 0.001, and ****P < 0.0001.

To confirm the impact of CCR7 functionality on monocyte maturation into macrophages and the effect of PGE_2_ treatment on this process, we performed chemotaxis assays using these cells and CCR7 ligands (Figure 7). First, Mono Mac-1 and MDMM-1 cells were incubated for 24 h in the presence or absence of PGE_2_ and evaluated for chemotaxis in response to 300 ng/mL CCL19 or CCL21. The differentiation of Mono Mac-1 cells significantly decreased their cellular responsiveness to both CCR7 ligands (Figure 7(a)); however, the chemotaxis of MDMM-1 cells increased upon treatment with PGE_2_, which supports the flow cytometry and RT-PCR results. In the case of MDMs, no specific migration was observed compared to that in freshly isolated blood monocytes (Figure 7(b)), confirming the absence of functional CCR7 receptors on the MDMs cell surface. Moreover, PGE_2_ failed to induce specific migration in MDMs, as was suggested by the flow cytometry results. To confirm the specificity of the observed migration, we incubated cells with a blocking antibody against human CCR7 for 10 min at room temperature prior to the migration assays [16]. This treatment completely abolished cell-specific migration in response to the CCL19 and CCL21 (data not shown).

## 4. Conclusion

In response to injury or infection, blood monocytes migrate to tissues to neutralize and eliminate potentially injurious stimuli. Depending on the inflammatory milieu and pathogen-associated pattern recognition receptors, monocytes may differentiate into inflammatory macrophages or DCs [28–30]. Monocytes can first differentiate into immature DCs and then mature upon exposure to antigens while changing their surface expression of chemokine receptors. Indeed, maturing DCs are known to downregulate CCR1, CCR5, and CCR6 expression and upregulate CCR7 expression (see review [31]). As CCR7 expression is not sufficient to ensure the migration of mature DCs in response to CCL19 and CCL21 [19, 32], CCR7 function is dependent on the presence of costimulatory signals such as PGE_2_ [17, 19]. Alternatively, monocytes can also differentiate into macrophages, long-lived cells that develop specialized functions such as phagocytosis. After phagocytosis, it has been postulated that macrophages will either migrate to the lymphoid organs or die by apoptosis at the inflammatory focus [33, 34]. However, it has recently been demonstrated that inflammatory macrophages, in addition to neutrophils, die via apoptosis during the late stages of resolving a proinflammatory insult [35]. 

In B cells, CCR9 and CCR10 expression permits the homing of immunoglobulin A plasma cells to mucosal tissues [36, 37], whereas CXCR3 and CXCR4 mediate the migration of IgG+ plasma cells toward inflamed tissues and bone marrow [38–40]. CXCR4 upregulation in T cells is implicated in the migration of leukocytes [41]. However, in bone marrow myeloid cells, CXCR4 downregulation is essential to promote their mobilization from bone marrow to the peripheral blood [42]. In addition, in DCs, PGE_2_ has been reported to synergize with TNF-*α* to promote CCR7 expression and the chemotactic responsiveness of DCs to CCL19 and CCL21 in lymph nodes [17–19]. These selected examples illustrate that chemokine receptor expression, together with their natural ligands, is involved and essential in the regulation of cellular migration. Recently, monocytes were shown to express CCR7 in the presence of the proinflammatory molecule PGE_2_, which is required to ensure migration in response to CCR7 ligands [16]. In the current study, we determined whether monocyte differentiation and the presence of the immunomodulatory molecule PGE_2_ had an effect on CCR7 expression and CCR7-dependent migration. Our results provide evidence that unpolarized blood macrophages do not express functional CCR7, even in the presence of PGE_2_ while polarized M1 macrophages have been shown to upregulate CCR7 expression [43]. We used the Mono Mac-1 cell line and freshly isolated blood monocytes that were differentiated into a macrophage-like cell phenotype (Figures 1 and 2). In both cases, we observed downregulation of CCR7 expression (Figures 3 and 6) and CCR7-specific migration (Figure 7). Further, we observed that the addition of PGE_2_ did not modulate CCR7 mRNA transcription, expression, and functionality in MDMs (Figures 5, 6, and 7). The results obtained using the cell line were different from those obtained using freshly isolated human blood monocytes/macrophages. In MDMM-1 cells, CCR7 expression and functionality were upregulated in response to PGE_2_. Similar results were found using THP1 cells, a human monocytic cell line. In that study, CCR7 expression in THP1-derived macrophages was increased following activation of the A_2A_ adenosine receptor [44]. The phenotypic differences between cell lines and human blood monocytes/macrophages could be explained by the tumor origins of both Mono Mac-1 and THP1 cells [45]. 

Our results suggest that human blood macrophages even in the presence of PGE_2_ do not migrate in response to the CCR7 natural ligands CCL19/CCL21. This reflects in vivo migration patterns because the collection of lymph through cannulation reveals that the major myeloid cell type to enter lymphatic vessels is antigen-presenting DCs rather than classical macrophages [7, 46]. Accordingly, monocyte-derived cells that leave acute inflammatory sites have a DC phenotype [47], and these cells migrate to the T-cell zone of the lymph node [47], which is rich in DCs but not in macrophages [48]. By contrast, little is known regarding macrophage migration. Migration of activated macrophages from the peritoneum to the lymphatics is accelerated by MAC-1, whereas early monocyte accumulation or subsequent redistribution within the peritoneum appears unaffected by this integrin [49].

Our findings are relevant to plaque formation in atherosclerosis because in this chronic inflammatory disease, the migratory process associated with resolution is impaired and macrophages accumulate in plaques, contributing to the build-up of necrotic pools [50]. Indeed, it has been demonstrated that mouse circulating monocyte subsets labeled with fluorescent latex beads did not emigrate out of plaques during disease progression [50, 51]. Restoration of monocyte-derived cell migration out of plaques could be an effective means to stimulate atherosclerosis disease regression. Moreover, some authors postulate that lipid-derived signals such as prostaglandins are strong candidates for impairing migration, and oxidized phospholipids along with lipoprotein A are disease-relevant mediators that may shift the fate of monocyte-derived cells to a more sessile phenotype [50, 52, 53]. Restoration of monocyte-derived cell migration out of plaques could be an effective means to stimulate atherosclerosis disease regression.

**Figure 8:**
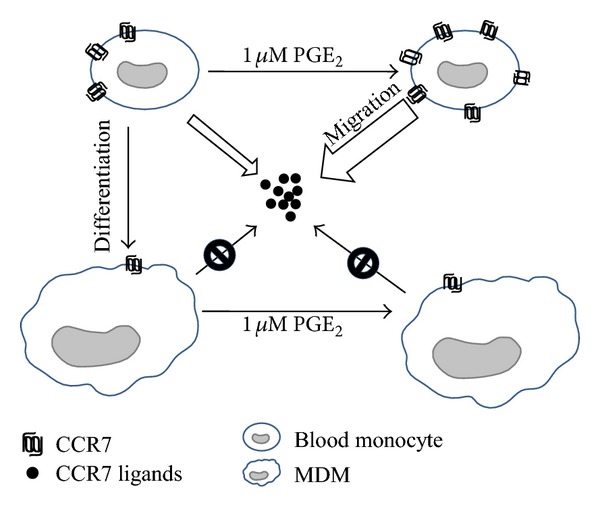
Schematic depicting CCR7 expression and functionality on human monocytes and MDMs.

In summary, we demonstrated that monocyte differentiation into macrophages directly modifies migratory behavior via CCR7 (Figure 8). Thus, our results suggest that human monocytes express CCR7 as shown with DCs, and T cells can, whereas unpolarized macrophages do not express CCR7 even in the presence of PGE_2_. Both MDMM-1 and MDM cells lost the functional capacity to migrate in response to the CCR7 ligands CCL19 and CCL21. However, we cannot state conclusively that the migratory behavior of monocytes and macrophages is due solely to CCR7 expression. The implication of other chemokine receptors and their regulation by prostaglandins should be investigated further.
